# Modular motor control of the sound limb in gait of people with trans-femoral amputation

**DOI:** 10.1186/s12984-019-0616-7

**Published:** 2019-11-06

**Authors:** Cristiano De Marchis, Simone Ranaldi, Mariano Serrao, Alberto Ranavolo, Francesco Draicchio, Francesco Lacquaniti, Silvia Conforto

**Affiliations:** 1 Department of Engineering, University Roma TRE, Roma, Italy; 2 Department of Medico-Surgical Sciences and Biotechnologies, University of Rome Sapienza, Roma, Italy; 3 Rehabilitation Centre, Policlinico Italia, Roma, Italy; 4 Department of Occupational and Environmental Medicine, Epidemiology and Hygiene, INAIL, Roma, Italy; 5 Department of Systems Medicine and Centre of Space Biomedicine, University of Rome Tor Vergata, Roma, Italy; 6 Laboratory of Neuromotor Physiology, IRCCS Santa Lucia Foundation, Roma, Italy

**Keywords:** Trans-femoral amputation, Lower limb prosthesis, Muscle synergies, Gait, sEMG, Modular motor control

## Abstract

**Background:**

The above-knee amputation of a lower limb is a severe impairment that affects significantly the ability to walk; considering this, a complex adaptation strategy at the neuromuscular level is needed in order to be able to move safely with a prosthetic knee. In literature, it has been demonstrated that muscle activity during walking can be described via the activation of a small set of muscle synergies. The analysis of the composition and the time activation profiles of such synergies have been found to be a valid tool for the description of the motor control schemes in pathological subjects.

**Methods:**

In this study, we used muscle synergy analysis techniques to characterize the differences in the modular motor control schemes between a population of 14 people with trans-femoral amputation and 12 healthy subjects walking at two different (slow and normal self-selected) speeds. Muscle synergies were extracted from a 12 lower-limb muscles sEMG recording via non-negative matrix factorization. Equivalence of the synergy vectors was quantified by a cross-validation procedure, while differences in terms of time activation coefficients were evaluated through the analysis of the activity in the different gait sub-phases.

**Results:**

Four synergies were able to reconstruct the muscle activity in all subjects. The spatial component of the synergy vectors did not change in all the analysed populations, while differences were present in the activity during the sound limb’s stance phase. Main features of people with trans-femoral amputation’s muscle synergy recruitment are a prolonged activation of the module composed of calf muscles and an additional activity of the hamstrings’ module before and after the prosthetic heel strike.

**Conclusions:**

Synergy-based results highlight how, although the complexity and the spatial organization of motor control schemes are the same found in healthy subjects, substantial differences are present in the synergies’ recruitment of people with trans femoral amputation. In particular, the most critical task during the gait cycle is the weight transfer from the sound limb to the prosthetic one. Future studies will integrate these results with the dynamics of movement, aiming to a complete neuro-mechanical characterization of people with trans-femoral amputation’s walking strategies that can be used to improve the rehabilitation therapies.

## Introduction

The above knee amputation is a severely invasive surgery that may be needed as a consequence of various causes such as vascular diseases, trauma or cancer [1]. After the surgery, people with trans-femoral amputation have to undergo a rehabilitation phase, in order to gain the ability to walk safely with a prosthetic device [2–4]. During the rehabilitation process, people with amputation must adapt their walking pattern to their new physical conditions and this adaptation may result in changes in the way the central nervous system (CNS) controls the movement. Considering this, a correct understanding of the strategies with which the CNS controls the musculoskeletal system in prosthetic gait can help with the design of advanced prosthetic devices and more efficient rehabilitation techniques.

In this kind of patients movement kinematics has been analysed in detail before [5], while surface electromyography (sEMG) has been used to assess changes in muscle activation only in a small set of studies dealing with gait and stair ascending [6, 7]; in addition, a complete characterization of the coordination of lower limb muscles in people with trans-femoral amputation is still missing. Such an analysis can be used to define some quantitative indicators of motor performances, so helping in guiding rehabilitation therapies.

Previous sEMG studies have shown that the most significant differences in muscular activity of both legs are found during the swing phase of the prosthetic limb (i.e. when all the body weight is on the sound leg), independently from the kind of prosthetic device [7]. Considering these results, a quantitative analysis of muscle activity during a gait cycle could underline some peculiar characteristics that can be used to determine objectively the motor performance of people with amputation.

It has been demonstrated that muscular activity in walking can be well represented by the activation of a small set of motor modules (*muscle synergies*) in healthy subjects [8–10]. Current theories suggest that the CNS controls and activates synergies depending on the particular task and on subtask constraints [11, 12], and some studies have proved that same motor modules are active in different motor tasks, suggesting that the composition of each muscle synergy reflects a spatial functional organization of the neuromuscular control at the CNS level [13]. Due to the fact that each motor module is responsible for a particular biomechanical function, the analysis of the spatial composition and the time activation profiles of muscle synergies may help with the functional characterization of movement [11, 14–16]. Muscle synergy analysis can provide valuable information for the neuro-mechanical characterization of movement, being able to model motor learning, motor adaptation and motor impairment after neurological damage [17]. Synergy analysis on post stroke patients has shown that the level of biomechanical impairment is correlated with the motor coordination complexity, so that subjects with lower biomechanical capacity typically exhibit a lower number of muscle synergies [18, 19]. These results suggest that neurological impairments affect the complexity of muscle coordination and modular control. For this reason, the analysis of muscle synergies has been proposed as a quantitative means for assessing the level of motor impairment and as a rehabilitation tool in the case of neurological pathologies [17, 20].

However, modular control of gait in people with trans-femoral amputation has not been analysed yet. In this study muscle synergies analysis techniques are applied to quantitatively assess the control strategies underlying walking with a prosthetic knee. Amputation of a lower limb is a biomechanical impairment, which implies alterations in the gait patterns and muscle coordination of a different nature with respect to neurological pathologies; as a consequence, we hypothesize that no significant changes in the coordination complexity and the composition of synergy vectors are present. Time activation coefficients, in contrast, are expected to give information on the changes in the control schemes for these patients, potentially describing alterations in the walking biomechanics.

The aim of this study is to analyse the spatio-temporal structure of the muscle synergies in patients with a trans-femoral amputation and to test the aforementioned hypotheses; for this reason, muscular coordination of the patients has been compared with that of a control group walking either at a comparable or self-selected speed, in order to disentangle potential effects of the intrinsic slower pace in people with trans-femoral amputation.

## Materials and methods

### Participants

Participants in this study included 14 subjects with a mono-lateral trans-femoral amputation (50 ± 14 years old) and 12 age matched healthy subjects (53 ± 8 years old). None of them had previous history of neurological pathologies and all the amputations were caused by traumatic events. Patients were experienced users (able to walk safely with a prosthetic knee for more than 1 year) of microprocessor controlled (C-Leg or Genium, Ottobock) knee prostheses. Details for the single subjects involved in the study can be found in Table 1.

**Table 1:** Age, Height, Weight and Walking speed for patients and control subjects

Patients						Control subjects					
Subject	Prosthetic Knee	Age (y.o)	Height (cm)	Weight (kg)	Speed (m/s)	Subject	Age (y.o.)	Height (cm)	Weight (kg)	Preferred speed (m/s)	Slow speed (m/s)
1	C-Leg	56	175	72	0.93	1	44	170	72	1.05	0.83
2	C-Leg	74	170	85	0.76	2	55	183	75	1.16	1.03
3	C-Leg	72	163	87	0.80	3	64	165	70	0.98	0.85
4	C-Leg	52	178	115	0.79	4	52	178	70	1.24	0.89
5	C-Leg	53	183	67	0.82	5	45	189	88	1.28	0.69
6	C-Leg	39	175	95	0.75	6	55	180	78	1.33	0.83
7	C-Leg	36	170	70	1.17	7	47	174	85	1.33	1.02
8	Genium	49	178	100	0.82	8	47	188	90	1.18	0.78
9	Genium	29	172	65	1.12	9	51	176	75	1.16	0.87
10	Genium	59	170	80	0.97	10	52	168	80	1.44	1.13
11	Genium	44	192	103	1.16	11	71	174	79	1.41	1.00
12	Genium	48	174	90	1.08	12	60	168	77	1.46	1.03
13	Genium	69	175	76	0.45						
14	Genium	30	178	95	0.98						

The whole study was approved by the local ethical committee (Rome branch of the INAIL Prosthesis Center, at the CTO “A. Alesini” in Rome) and was carried out in accordance with the principles of the declaration of Helsinki.

### sEMG and kinematic recordings

sEMG data were recorded from 12 muscles of the sound limb in subjects with a trans-femoral amputation and the right leg in the control group: *rectus femoris* (RF), *vastus lateralis* (VL), *vastus medialis* (VM), *gluteus medius* (GM), *tensor fasciae latae* (TFL), *semitendinosus* (ST), *biceps femoris* (BF), *tibialis anterior* (TA), *peroneus longus* (PL), *soleus* (SOL), *gastrocnemius lateralis* (GL) and *gastrocnemius medialis* (GM). sEMG signals were acquired in a bipolar configuration at a sampling frequency of 1000 Hz and digitized at 16 bits with a BTS FREEEMG1000 system; electrodes were placed on the skin according to the SENIAM standard [21]. Kinematic data from both the lower limbs were recorded via a stereophotogrammetric system (BTS SMART-DX 6000) at a rate of 340 Hz and synchronized with sEMG data. A Davis marker set [22] was used for full body kinematic recording; these data were used in the present study for the calculation of gait speed and gait events and for the computation of average profiles for the hip, knee and ankle flexion-extension angles. Ground reaction forces (Kistler 9286AA) were recorded (sampling frequency 680 Hz) by means of two force platforms included in the walkway.

Kinematic and kinetic data were used only as a qualitative reference for understanding the biomechanical meaning of the synergy-based results.

### Experimental protocol

All the experimental procedure was performed on a 9 m walkway; the two force plates were hidden in the central part of the walkway so that all the subjects were not aware of the presence of the platform. Subjects with Trans-Femoral amputation (**TF**) were asked to walk from one side of the walkway to the other side with a self-selected comfortable speed (0.9 ± 0.2 m/s). Each subject performed ten walking repetitions. Healthy control subjects performed the same task at two different speeds, namely self-selected preferred (**C**_**SS**_ group, 1.2 ± 0.1 m/s) and self-selected slow (**C**_**SL**_ group, 0.9 ± 0.1 m/s) speed. Only the central strides, in which the heel strike and toe off events could be detected by the corresponding reflective markers, were used for further analysis; this resulted on 7.9 ± 1.2 (mean ± SD) complete gait cycles per subjects belonging to each group.

The two different walking speeds for control subjects were needed in order to separate any speed-dependent feature of the control strategies from actual characteristics of people with a trans-femoral amputation; for this reason, in this work the control population walking at the two different speeds will be considered as two separate groups, one of which (C_SL_) is speed matched with the TF group.

### Data preprocessing

Kinematic data were used to detect heel strike (HS) and toe off (TO) events of both the sound (the one equipped with sEMG sensors and considered as the reference leg) and the prosthetic leg (non-reference leg). For controls the reference leg is the one equipped with sEMG sensors, i.e. the right leg.

HS and TO were used to define, for each stride, four sub-phases as follows:

- First double support phase (**DS1**), defined as the time period going from the reference leg HS to the upcoming non-reference leg TO.
- Single Stance (**Stance**), defined as the time period going from the non-reference leg TO to the non-reference leg HS (i.e. the single support phase of the reference leg).
- Second double support phase (**DS2**), defined as the time interval going from the non-reference leg HS to the reference leg TO.
- Swing phase (**Swing**), defined as the swing of the reference leg, going from the reference leg TO to the upcoming reference leg HS.

sEMG data were bandpass filtered between 35 and 450 Hz (4th order, Butterworth), and the sEMG envelope was extracted with the adaptive algorithm described in [23]. This algorithm exploits information theory to find a sample by sample optimal RMS window for the envelope estimation; using this algorithm ensures that fast changes in sEMG activity are correctly followed by the filter, while still maintaining an optimal performance when the sEMG amplitude is slowly varying.

sEMG envelope amplitude within each stride was normalized at the median value of the peaks from all the analysed gait strides.

After envelope extraction, time scales were normalized by interpolating the envelope within the previously defined sub-phases of the walking cycles on a fixed number of samples (DS1 - 20samples, Stance - 80samples, DS2 - 20samples, Swing - 80samples), so to obtain a 200-points time scale normalization of each stride. An average activation profile for each subject and each muscle was then obtained from the time-normalized envelope.

An average profile for hip and knee flexion-extension angles and for ankle dorsi-plantar flexion has been defined for each subject using the same time scale normalization procedure described before. In the same way, a characteristic, 3-components ground reaction forces profile has been extracted from a subset of the trials from each subject. Those curves will be used as a qualitative support for the neuromechanical interpretation of the synergy-based results.

### Extraction of muscle synergies

Muscle synergies were extracted by applying a non-negative matrix factorization (NNMF) algorithm to the *12 x (N*_*S*_*)* matrix containing the sEMG envelopes before time scale normalization, where N_S_ is the number of samples for each signal. This procedure was adopted in order to avoid any effect of gait phase differences on muscle synergy extraction. For a particular number of synergies *N*_*syn*_, NNMF approximates the envelopes matrix M by the product of two matrices W and H, where W is the *12 x N*_*syn*_ matrix containing the synergy vectors and H is the *N*_*syn*_ *x (N*_*S*_*)* matrix of the time activation coefficients, following the synchronous muscle synergy model (i.e. fixed spatial components):

$$ {M}_{\beta }(t)=\sum \limits_{i=1}^{N_{syn}}{W}_{i,\beta }{H}_i(t) $$

where *β* represents each muscle. NNMF was applied with a sparse initialization in order to increase muscle synergy identification accuracy [24].

After extraction, each synergy vector (i.e. each column of the W matrix) was normalized to its norm, and the corresponding time activation coefficient was scaled of the same quantity to keep the reconstruction *W x H* unchanged. The synergy vector contains the relative contribution of each muscle to each synergy, while the time activation coefficients provide information regarding the recruitment of a group of muscles within the gait cycle.

The number of synergies N_syn_ to be extracted from each subject was selected based on the analysis of the variance accounted for (VAF) for the whole envelope matrix reconstruction. The minimum number of synergies for which the global VAF values exceeds 90% was selected as the correct one. The nearest integer greater than the mean value of the number of synergies for all subjects in each population was selected as the number of synergies to be extracted from the whole group for comparison between healthy and pathological set of synergies. After the definition of the characteristic N_syn_ for each population, the same number of muscle synergies was extracted from each subject.

Synergy vectors W for each subject were ordered to maximize cosine similarity (i.e. the normalized dot product) between the W of the different individuals, and a characteristic set of W for each population was defined as the average of the ordered sets. Average synergy vectors coming from the C_SS_ group were taken as the reference W (W_ctrl_). Similarity between the average set of W vectors has been quantified using the normalized dot product.

### Cross-validation procedure

A cross-validation procedure was used to assess if the representative vectors W_ctrl_ are able to reconstruct muscle activity in all the subjects from each group.

This procedure is carried on by applying a non-negative reconstruction (NNR) technique to the envelope matrix of each subject, by keeping W_ctrl_ fixed and updating the (*α*, *μ*) element of the H matrix following the update rule

$$ {H}_{a\mu}\leftarrow \frac{{\left({W}^TM\right)}_{a\mu}}{{\left({W}^T WH\right)}_{a\mu}} $$

Where α and μ indicate the rows and columns, respectively, of the corresponding matrices. VAF values for the reconstruction are evaluated and compared with the 95th percentile of the distribution of VAF values coming from different reconstructions with random synergy vectors; these vectors were obtained by random shuffling the components of the original W matrix within each synergy. If the reconstruction VAF value is higher than the threshold so defined, W_ctrl_ is hypothesized to be representative of the motor control strategies for that particular subject.

### Activation coefficients parameters

Once having defined the equivalence of the W_ctrl_ for all the groups, the time coefficient analysis was carried out on the reconstructed profiles relative to the aforementioned set of synergies. After reconstruction, H coefficients have been normalized to the time scale described before. For each subject, the mean activation profile was then calculated as the average of the time-normalized H profiles across cycles.

From the mean activation profiles of each subject, we evaluated an indicator of the activity in each phase as the sum of the corresponding samples (i.e. 0–20 for **DS1**, 21–100 for **Stance**, 101–120 for **DS2** and 121–200 for **Swing**). In addition, as a qualitative measure of the time localization of the synergy activity within the gait cycle, we calculated the centre of activity (CoA) for each H profile as defined in [25].

### Statistical analysis

All the statistical differences in the time activation parameters related to the H coefficients (i.e. activity in each gait sub-phase) were evaluated by means of a Kruskal-Wallis test with group as factors (TF, C_SS_ and C_SL_). The test on the activation parameters was carried out independently for each synergy and each sub-phase of the gait cycle (**DS1**, **Stance**, **DS2**, **Swing**). Post-hoc analysis was carried out using Bonferroni correction and statistical significance was set to *α* = 0.05.

Equivalence of N_syn_ was evaluated by means of a Fisher’s test, with statistical significance set to *α* = 0.05.

## Results

### Single muscles activation profiles

**Fig. 1:**
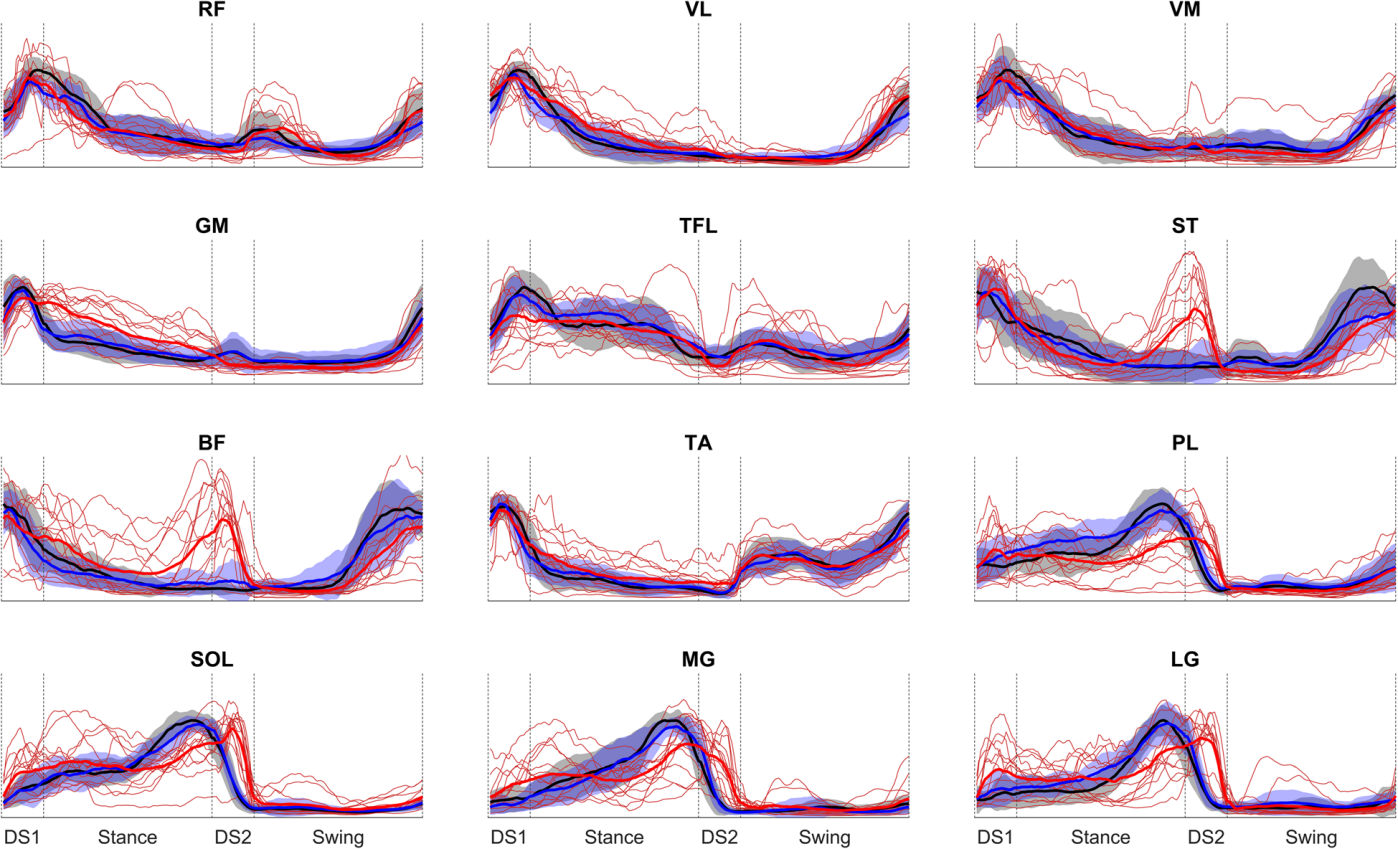
Mean activation profiles for each of the 12 muscles used in the study. Orange: single TF subjects; Black: C_SS_; Blue: C_SL_; Red: TF. Grey: normality band from the C_SS_ group. Blue: normality band from the C_SL_ group

Average muscle activation profiles for each muscle for the different groups are shown in Fig. 1. All the main qualitative differences in the average profiles are visible in the stance phase of the sound limb, particularly in the muscles belonging to the back side of the leg (i.e. ST, BF and the calf muscles). In general, the patients show a higher variability with respect to the control populations.

### Number and structure of synergies

The mean number of synergies able to reconstruct the activation of each population is higher than 3 for each of the three groups (details of the percentages of subjects characterized by 3, 4 or 5 synergies for each group is shown in Table 2); considering this, 4 synergies were extracted from each subject as the minimum number able to reconstruct the muscle activity in approximately the 90% of the subjects.

**Table 2:** Percentage of subjects requiring 3, 4 or 5 synergies for each group

	3 SYNERGIES	4 synergies	5 synergies
C_SS_	58%	42%	0%
C_SL_	58%	33%	9%
TF	43%	36%	21%

The Fisher’s test showed the equivalence of the number of synergies for the three groups (*p* = 0.58).

**Fig. 2:**
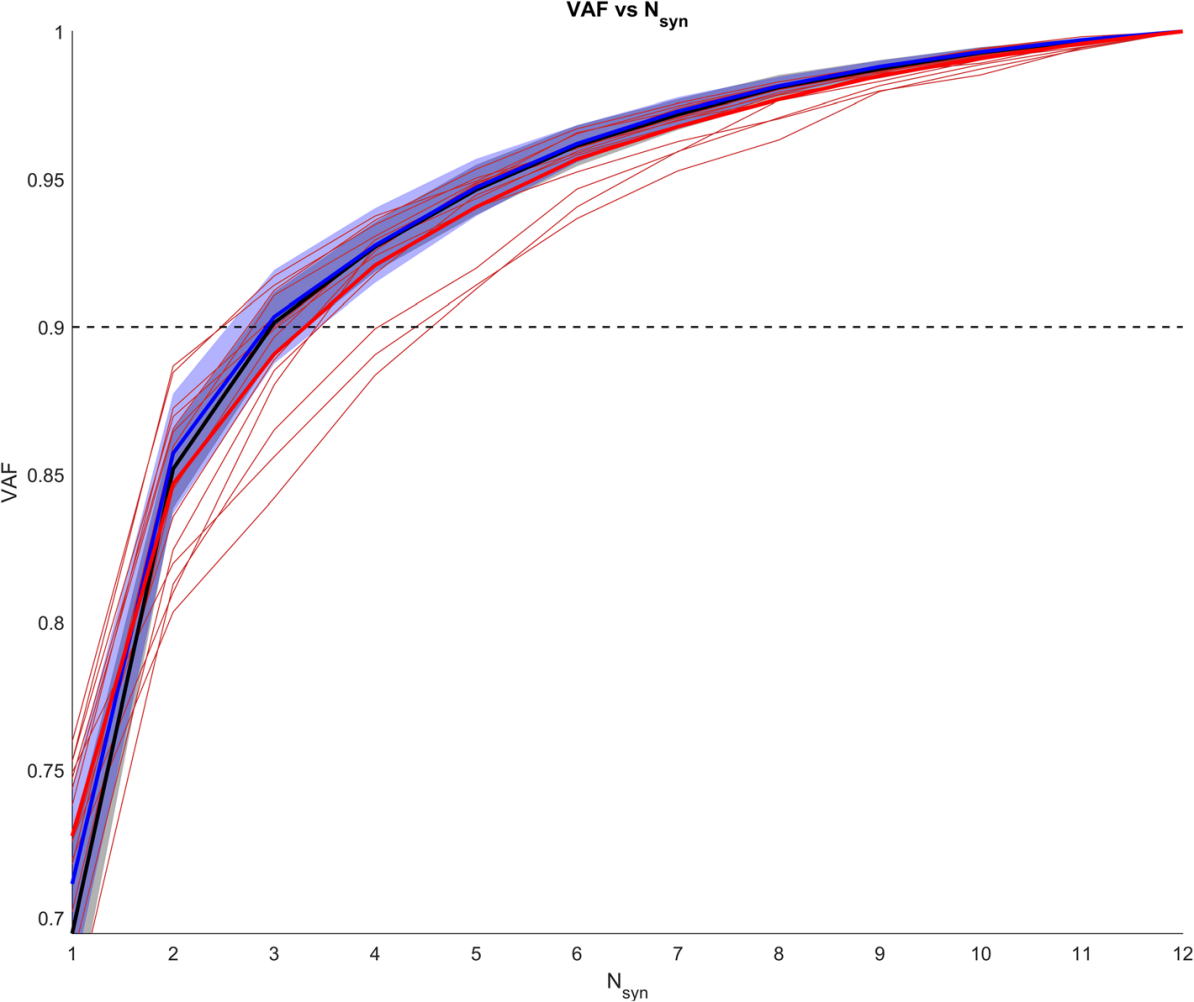
Average VAF vs Nsyn curve for the three groups (Black: C_SS_, Blue: C_SL_ and Red: TF) and single subject curves for the TF population (light orange). Grey: normality band from the C_SS_ group. Blue: normality band from the C_SL_ group

The mean VAF profiles for the three populations are shown in Fig. 2, together with the curves for each TF subject.

The VAF values for the extraction of 4 synergies from all subjects were: 0.93 ± 0.01 for C_SS_, 0.93 ± 0.01 for C_SL_ and 0.92 ± 0.02 for TF.

**Fig. 3:**
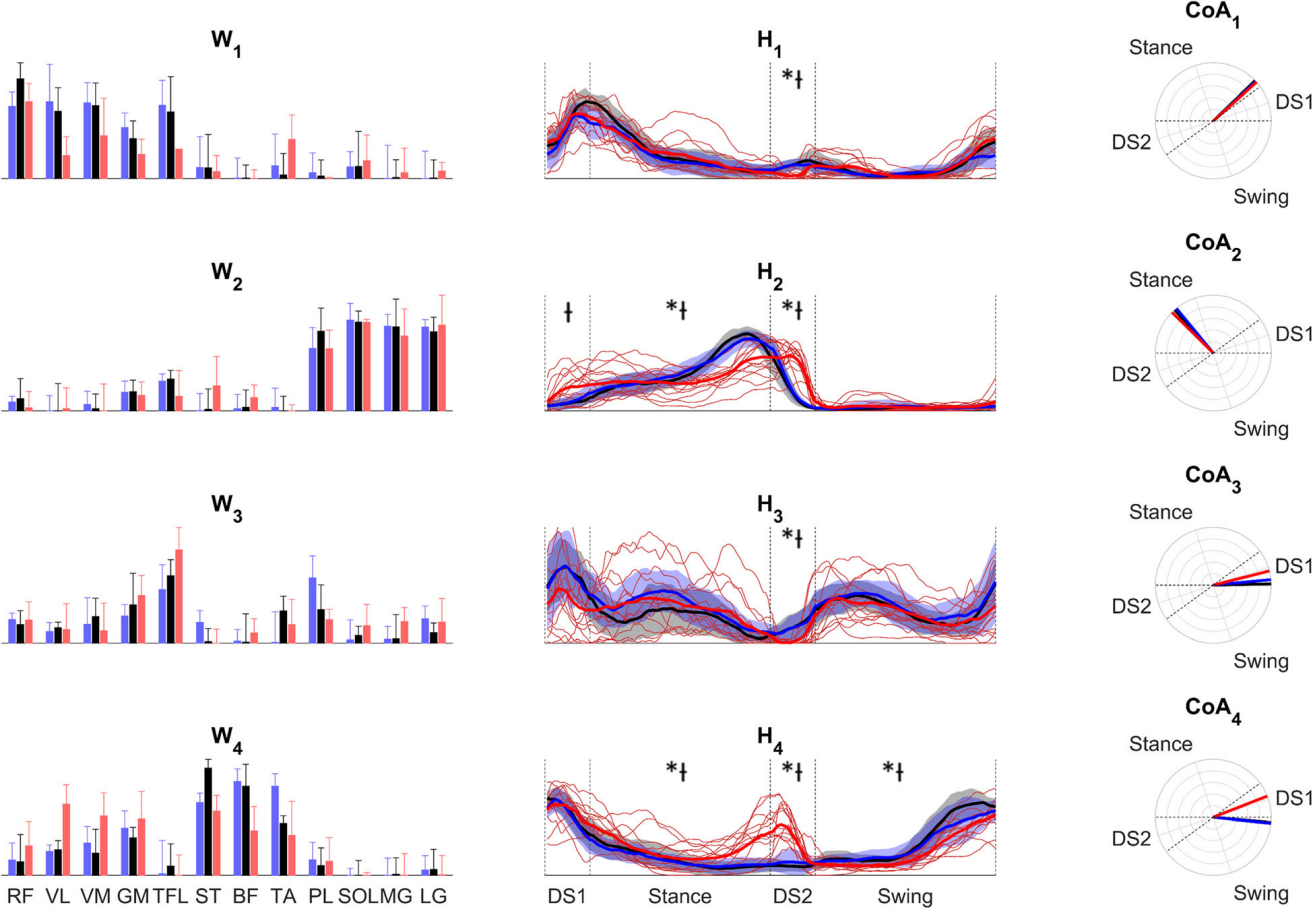
Left panel: characteristic W for each population; Center panel, activation coefficients coming from the reconstruction with W_ctrl_ (in black in the left panel) (*: difference between TF and C_SS_; Ɨ: difference between TF and C_SL_); Right panel: CoA values (360 degrees = 1 gait cycles). Black: C_SS_, Blue: C_SL_, Red: TF. Grey: normality band from the C_SS_ group. Blue: normality band from the C_SL_ group

The cross-validation procedure described in the methods section has shown how the W_ctrl_ synergies can reconstruct well the activation of C_SS_, C_SL_ and TF subjects (reconstruction VAF: 0.88 ± 0.02 for C_SS_, 0.88 ± 0.02 for C_SL_, 0.85 ± 0.03 for TF. All of them systematically higher from VAF values expected from chance). The average W vectors for each population are shown in the left column of Fig. 3. Cosine similarity values of the average synergies have been found to be systematically higher than 0.8 for each pair of corresponding W vectors (ranges 0.82–0.97 for C_SS_ vs TF, 0.80–0.98 for C_SL_ vs TF and 0.84–0.99 for C_SS_ vs C_SL_).

### Time activation profiles

Since the structure of the muscle synergies has not shown significant differences between the three groups, the analysis has been carried out on the features of the time activation profiles coming from the reconstruction with W_ctrl_ (Fig. 3, central column).

Significant differences during the four phases are marked in the central column of Fig. 3. The DS2 phase showed differences in the activation of all synergies (*H*_*1*_*: C*_*SS*_ vs *TF p = 0.003, C*_*SL*_ vs *TF p = 0.012; H*_*2*_*: C*_*SS*_ vs *TF p < 0.001, C*_*SL*_ vs *TF p = 0.001; H*_*3*_*: C*_*SS*_ vs *TF p = 0.010, C*_*SL*_ vs *TF p = 0.012; H*_*4*_*: C*_*SS*_ vs *TF p < 0.001, C*_*SL*_ vs *TF p = 0.001*); several other differences were present in the activity during DS1 (*H*_*2*_*: C*_*SS*_ vs *TF p = 0.020*), Stance (*H*_*2*_*: C*_*SS*_ vs *TF p = 0.026, C*_*SL*_ vs *TF p = 0.025; H*_*4*_*: C*_*SS*_ vs *TF p = 0.030, C*_*SL*_ vs *TF p = 0.015)* and Swing (*H*_*4*_*: C*_*SS*_ vs *TF p = 0.001, C*_*SL*_ vs *TF p = 0.026*)*.* In particular, TF subjects showed a lower activity of H_1_ and H_3_ during DS2 and of H_4_ during Swing, together with a higher activity of H_2_ during the two double support phases and of H_4_ during Stance and DS2. No differences have been found between C_SS_ and C_SL_.

The CoA values reported in the right column of Fig. 3 showed that the synergies 3 and 4 are characterized by a shift towards a different gait phase in TF with respect to C_SS_ and C_SL_.

**Fig. 4:**
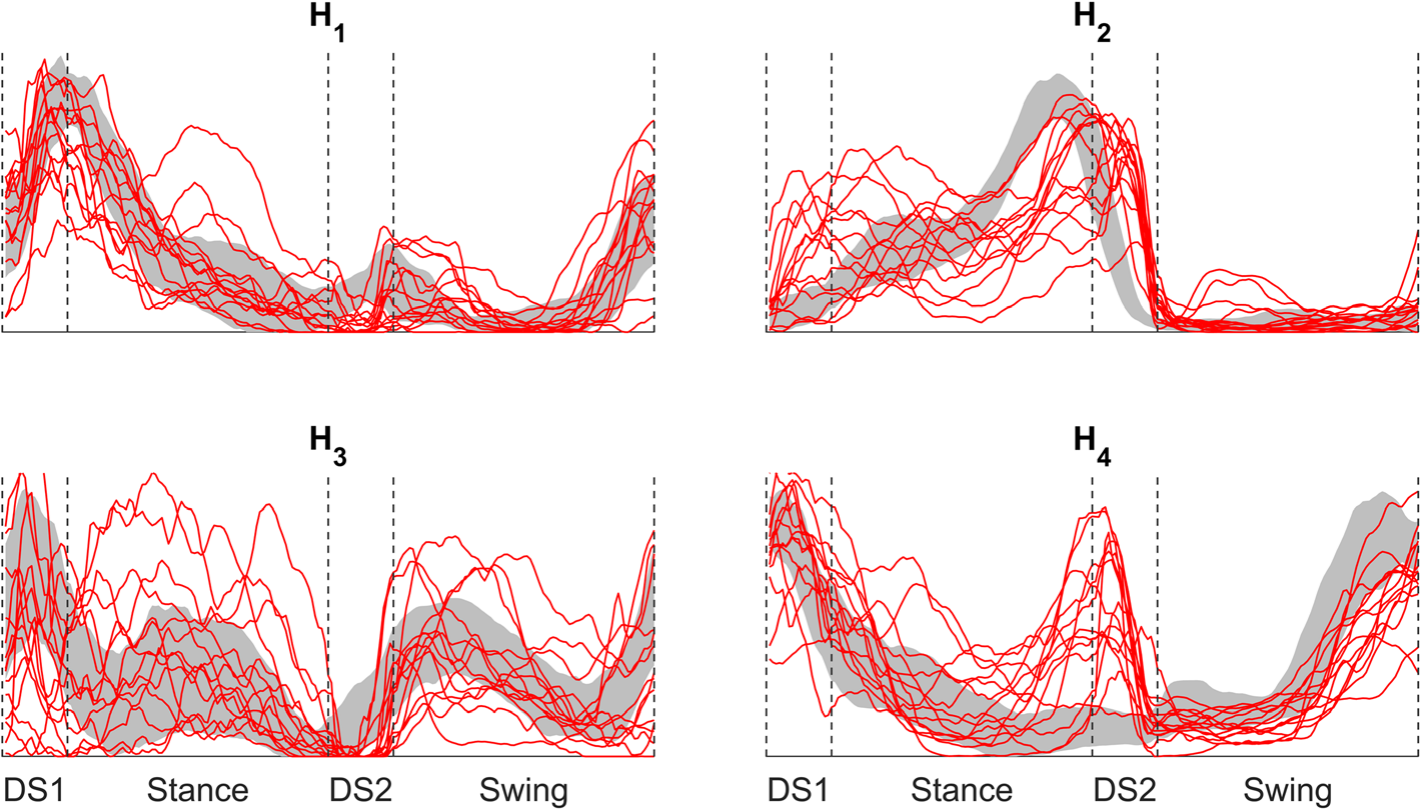
H profiles (average across cycles) for each TF subject (Red lines). Grey: normality band from the C_SS_ group. Blue: normality band from the C_SL_ group

A comparison between the normality band (mean ± standard deviation) relative to the C_SS_ subjects and all the TF profiles for the four average activation coefficients is shown in Fig. 4.

## Discussion

Results of this study prove that activation profiles of the sound limb muscles of people with trans-femoral amputation during gait can be well reconstructed by a set of four muscle synergies. Results also confirm that the complexity of muscle coordination (i.e. the number of underlying muscle synergies) is maintained after the trans-femoral amputation of a lower limb.

The cross-validation procedure and the dot product values show that the structure of the muscle synergies does not differ significantly from the one extracted from a population of control walking at two different speeds. In particular, results suggest that the general motor coordination schemes are not different from the case of non-pathological gait.

The invariance of the composition of muscular synergies confirms our hypothesis that the modular motor control strategy does not change as a consequence of an amputation. The composition of synergy vectors is indeed similar to the one extracted in other studies on human walking [8, 10, 18, 26]. Each of the four synergies is composed of muscles with a similar functional role during walking, in accordance with all the previous studies on modular motor control of gait; therefore, these results show that the basic biomechanical functions during gait are preserved after a trans-femoral amputation [8]. In particular, the four synergies have been proved to be responsible of the following functions during gait:

- W_1_ (Knee extensors and GM): mostly involved in weight acceptance and body weight support
- W_2_ (Calf muscles): involved in body weight support and propulsion before toe-off
- W_3_ (TFL with some minor contributions from knee extensors, TA and PL): responsible for the swinging movement of the leg and for the weight acceptance phase
- W_4_ (Hamstrings and TA): responsible for the late swing leg deceleration

The combination of the results of the cross-validation analysis and of the high cosine similarity between W vectors provides strong evidence that the spatial structure is equivalent in the three groups. The choice of selecting the characteristic W coming from the healthy subjects walking at a self-selected speed, instead of the other groups, has been made in order to fix the spatial structure that can be extracted from healthy and unconstrained (i.e. at a self-selected speed) gait. By doing so, it is possible to hypothesize that any difference related to the speed will be contained in the features of the time activation coefficients, providing an easier interpretation of any alteration in gait patterns.

**Fig. 5:**
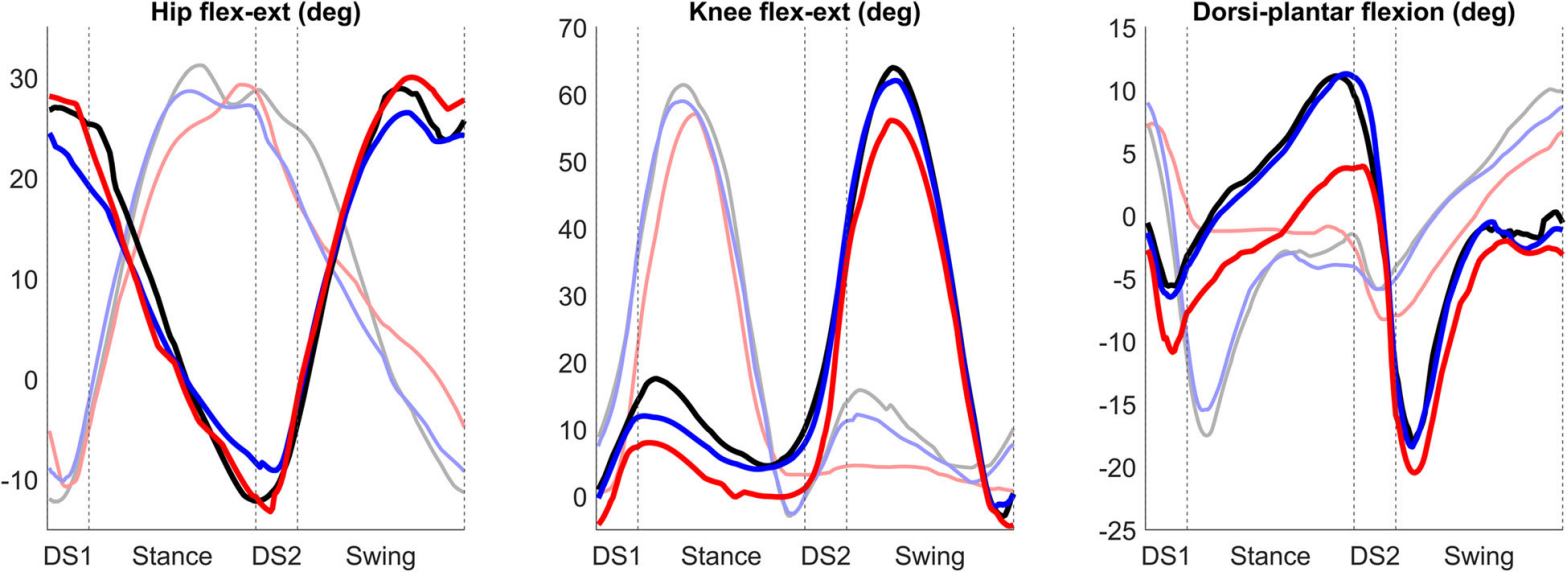
Mean profiles for the three flexion-extension angles for the reference leg. Black: C_SS_; Blue: C_SL_; Red: TF. Shaded colours: non-reference leg

**Fig. 6:**
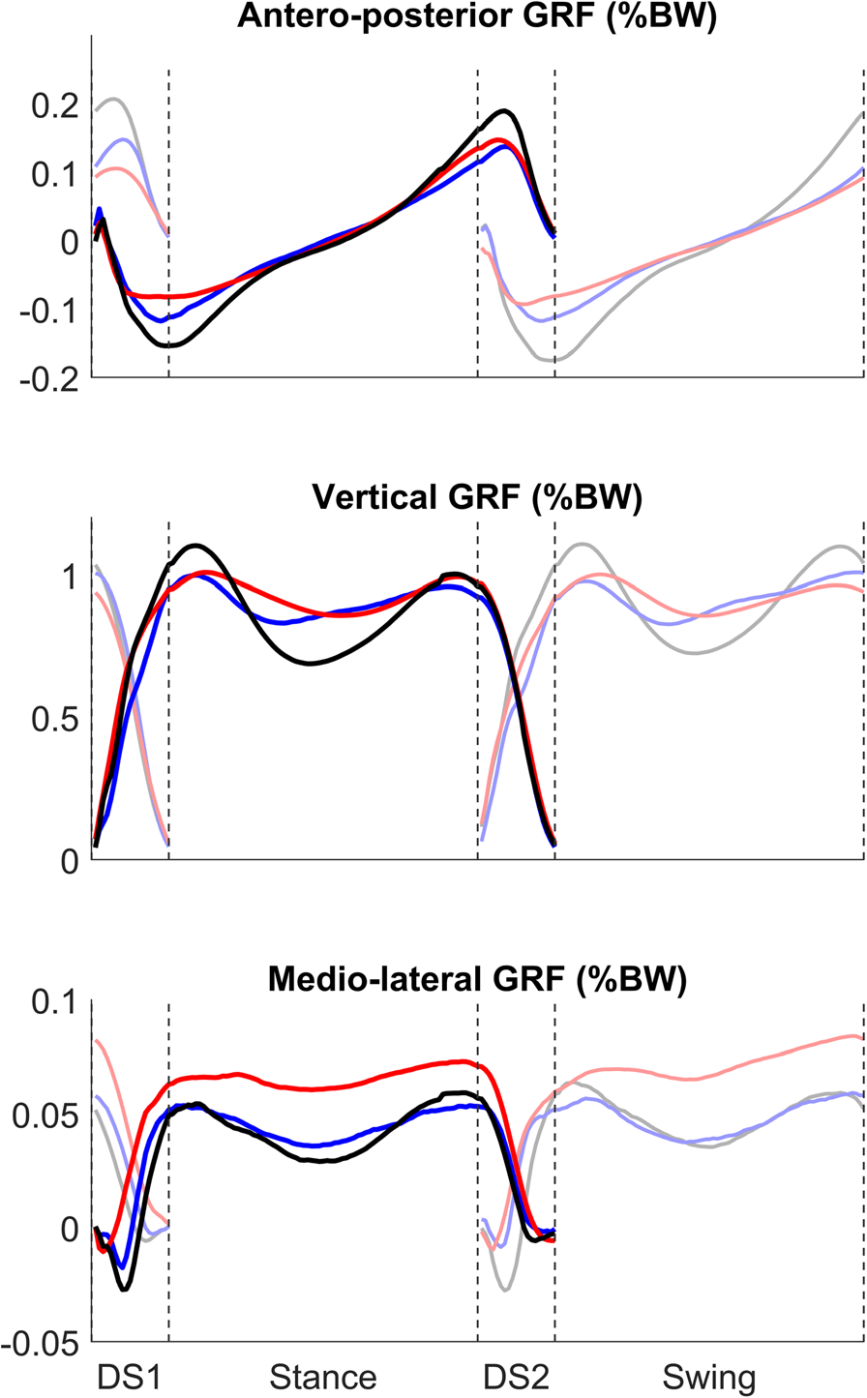
Mean profiles for the three components of the ground reaction forces for the reference limb. Black: C_SS_; Blue: C_SL_; Red: TF. Shaded colours: non-reference limb

The results presented before show how the most critical phase in gait of people with trans-femoral amputation is the second double support phase, corresponding to the weight transfer phase from the sound limb to the prosthetic one. In this portion of the gait cycle, all the muscle synergies showed a significantly different activity in people with trans-femoral amputation; this result is coherent with studies that investigated gait of this kind of subjects from a metabolic point of view using inverse dynamics, finding that the most energy demanding task in gait is the transfer of the body weight from each leg to the other [27]. From Figs. 5 and 6, reported here as a support for the interpretation of our results, one can notice that both limb kinematics (joint angles) and kinetics (ground reaction forces) have, on average, very similar profiles in amputees and control subjects during the second double support phase. In particular, the time changes of the vertical component of the ground reaction forces seem identical in the two groups of participants, indicating that the weight transfer from the sound limb to the prosthetic one was accomplished in a smooth manner also in amputees. Therefore, the significant changes of the time activation coefficients of the synergies in amputees during the weight transfer phase probably represent an efficient compensatory mechanism that develops in these subjects after extensive experience with the prosthesis. This hypothesis is supported by the fact that our average kinematic and dynamic profiles show features that are consistent with the ones reported before in literature [5].

The activation profile of the calf muscles module W_2_ shows a different activity in all those gait phases in which the sound limb is in contact with the ground, and this behaviour reflects the tendency to prolong the stance phase of the sound limb with respect to the prosthetic limb’s [7], using the ankle to reduce the effect of the body inertia. The different activity of this module, particularly during the first double support phase, can be the cause for the reduced dorsiflexion recorded for these patients; however, this behaviour has to be analysed in conjunction with the activity of the fourth module, as these two synergies could play the main role for the changes in the control strategies. This reduced dorsiflexion can also be the result of a decreased intact leg deceleration activity and the resulting greater hip flexion at the intact limb heel strike; this characteristic has been found before in literature [28] and can be an interpretation for the reduced activity of the fourth module in swing. Moreover, from a visual analysis of the activation patterns shown in Fig. 4, patients seem to be divided into two groups depending on the activation of the calf synergy at the beginning of the stance phase; this aspect could be analysed in future studies aiming to a complete neuro-mechanical characterization of prosthetic gait.

The shift towards the first double support phase of the centre of activity of the fourth synergy is mainly due to the presence of an additional peak of activity; given this, it is possible to conclude that its main role of decelerating the leg in late swing [8] is preserved, while an additional activation is required for some kind of neuromechanical compensation strategy, possibly involving an additional hip extension moment. Previous studies [29] have shown how an increased activity of the hip extensors during early stance can have a key role in compensating for the smaller propulsive activity of the prosthetic limb. The additional activity at the prosthetic limb initial contact has not been reported before; based on our results, however, it is possible to hypothesize that this contraction is needed to compensate for the smaller dorsiflexion during the intact limb stance, providing additional propulsion to the body before the prosthetic foot hits the ground.

For what concerns the shift in the third module centre of activity, this is not the consequence of an additional activation; instead, even if the CoA does not shift into a different phase, this characteristic can reflect in a synthetic way a different ratio of the activities of the synergy during the first double support and the single stance phases. This feature of gait in people with trans-femoral amputation can be the result of different, subject-specific stabilization mechanisms for the hip during the stance phase; hip stabilizer might work together with the second module in compensating for any differences in the ground reaction forces that are visible in the medio-lateral average profiles.

Since no statistical difference has been found between controls walking at different speeds, any difference between the patients and one of the two control groups can be interpreted as a typical sign of altered neuromuscular control in people with trans-femoral amputation. However, further statistical analysis, including a larger control groups walking at a wider range of speeds and the analysis of a larger number of strides, could reinforce these findings.

## Conclusions

In this study, we used muscle synergy analysis techniques to characterize the neuromuscular control strategies during people with trans-femoral amputation’ gait, by comparing muscle synergies extracted from a population of patients with the ones found in a control group walking at two different speeds. Our results showed that, although the complexity and the spatial structure of the modular motor control schemes are preserved after an amputation, some crucial differences can be found in the timing of the activation of muscle synergies. All the muscle synergies have shown different activities during the weight transfer phase from the sound to the prosthetic limb, suggesting that, from a neuromuscular point of view, this is the most critical phase of the gait cycle. The combination of these results with an investigation of the dynamics of movement can yield a complete characterization of people with trans-femoral amputation’s gait, so helping in guiding the rehabilitation strategies towards a solution that can improve the overall walking performance of the patients.

## Data Availability

The datasets generated and/or analysed during the current study are not publicly available due to clinical policy but are available from the corresponding author on reasonable request.

## Abbreviations

CNS: Central Nervous System
CoA: Centre of Activity
HS: Heel Strike
NNMF: Non-Negative Matrix Factorization
NNR: Non-Negative Reconstruction
sEMG: surface ElectroMyoGraphy
TO: Toe Off
VAF: Variance Accounted For
